# Supplementary material for: Predicting visual function by interpreting a neuronal wiring diagram
Source: Nature. 2024 Oct 2;634(8032):113–23. doi: 10.1038/s41586-024-07953-5 (PMC11446822; doi:10.1038/s41586-024-07953-5)
Supplement: Supplementary file 6 — CRF and ERF predictions for individual TmY4 and TmY9 cells. Analogous to Supplementary Data 3, but for TmY target types. Shown are the top four monosynaptic pathways, the strongest pathway passing through each of the top ten intermediary types (ranking from Extended Data Fig. 7), and the trisynaptic pathway Tm1–TmY–Dm3–TmY (see the section entitled Prediction of spatial normalization). [file 41586_2024_7953_MOESM6_ESM.zip › DataS4/TmY9qΓèÑ/720575940606240958.pdf]

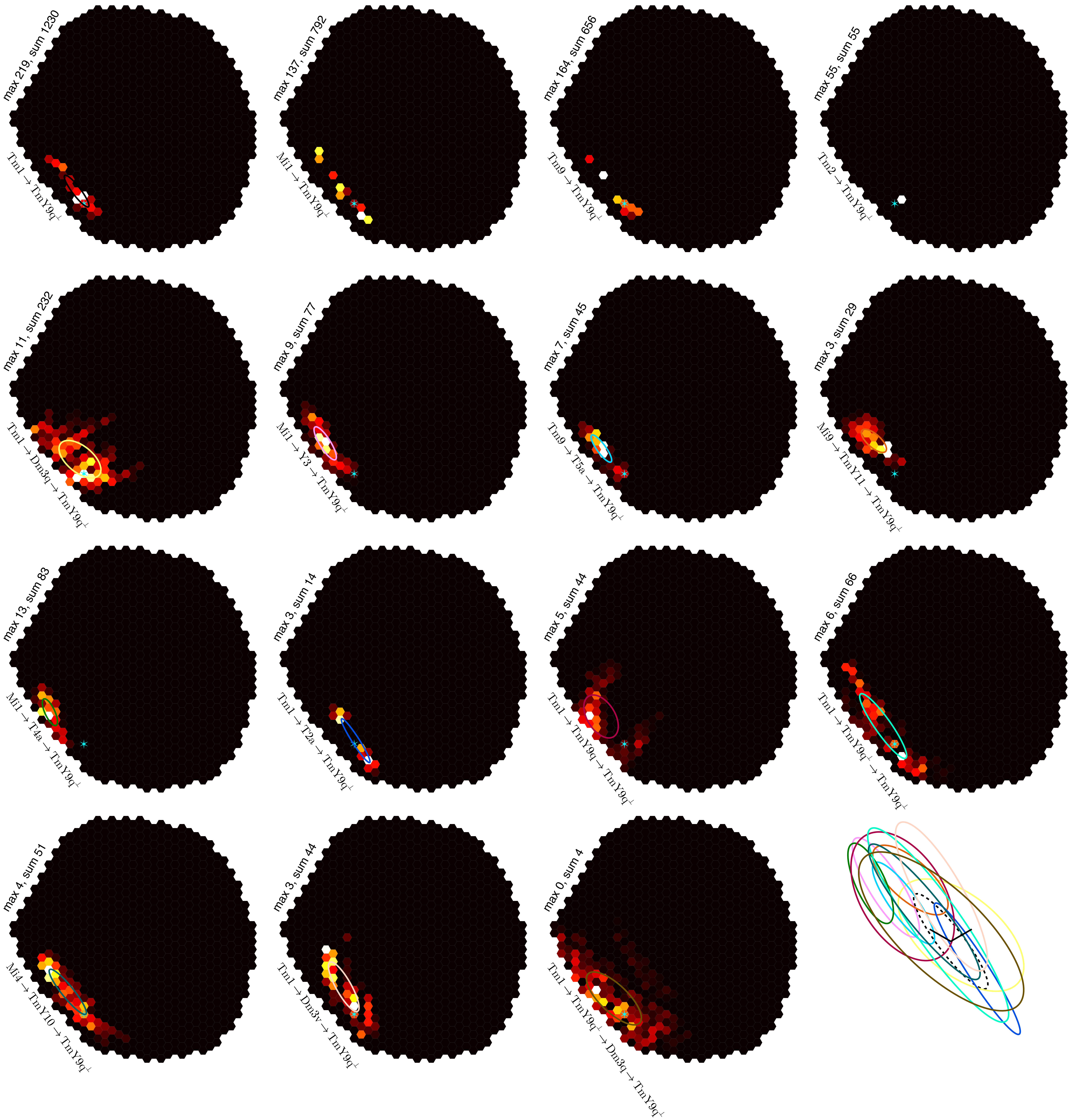
max 219, sum 1230
Tm1 → TmY9q⊥
max 137, sum 792
Mi1 → TmY9q⊥
max 164, sum 656
Tm9 → TmY9q⊥
max 55, sum 55
Tm2 → TmY9q⊥
max 11, sum 232
Tm1 → Dm3q → TmY9q⊥
max 9, sum 77
Mi1 → Y3 → TmY9q⊥
max 7, sum 45
Tm9 → T5a → TmY9q⊥
max 3, sum 29
Mi9 → TmY11 → TmY9q⊥
max 13, sum 83
Mi1 → T4a → TmY9q⊥
max 3, sum 14
Tm1 → T2a → TmY9q⊥
max 5, sum 44
Tm1 → TmY9q → TmY9q⊥
max 6, sum 66
Tm1 → TmY9q⊥ → TmY9q⊥
max 4, sum 51
Mi4 → TmY10 → TmY9q⊥
max 3, sum 44
Tm1 → Dm3v → TmY9q⊥
max 0, sum 4
Tm1 → TmY9q⊥ → Dm3q → TmY9q⊥
